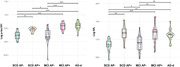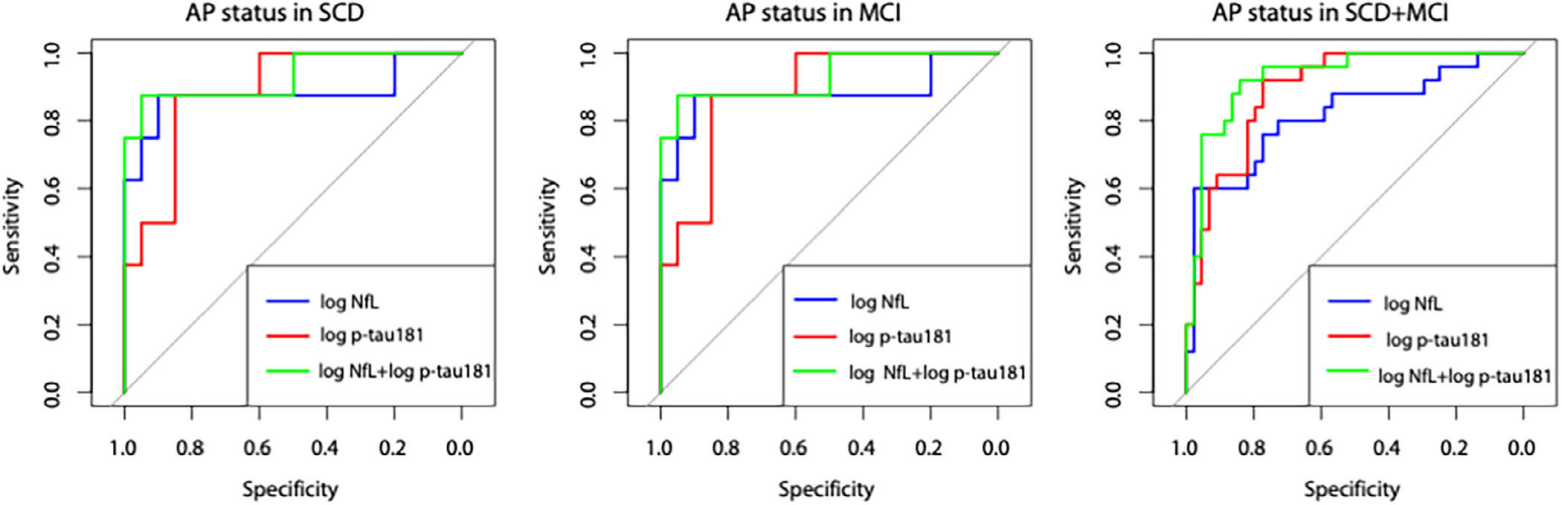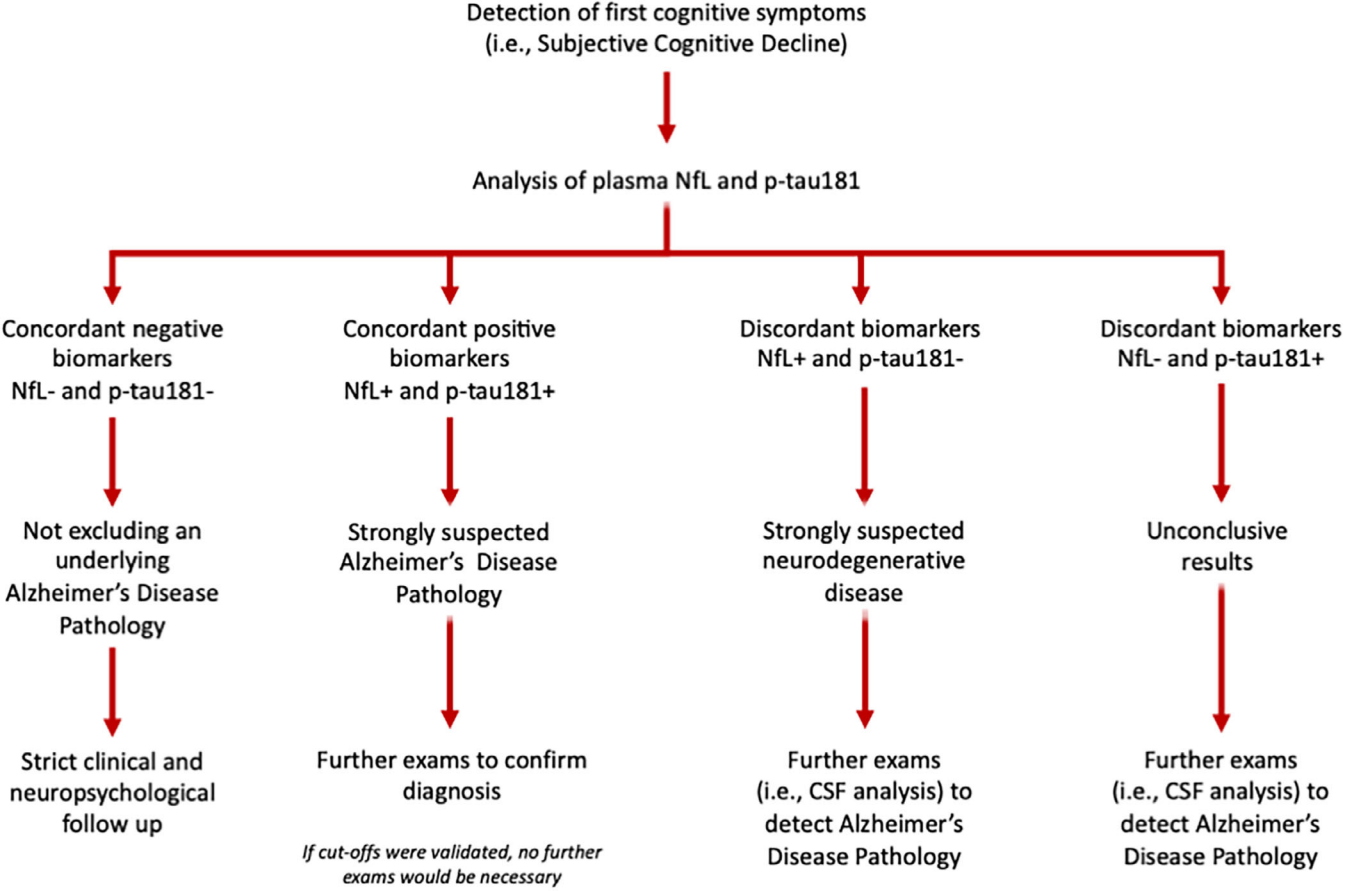# Combined use of plasma p‐tau 181 and neurofilament light chain in Subjective Cognitive Decline and Mild Cognitive Impairment: future perspective and clinical applicability

**DOI:** 10.1002/alz.085914

**Published:** 2025-01-09

**Authors:** Giulia Giacomucci, Salvatore Mazzeo, Assunta Ingannato, Chiara Crucitti, Silvia Bagnoli, Sonia Padiglioni, Giulia Galdo, Filippo Emiliani, Daniele Frigerio, Valentina Moschini, Carmen Morinelli, Sandro Sorbi, Benedetta Nacmias, Valentina Bessi

**Affiliations:** ^1^ University of Florence, Florence, Florence Italy; ^2^ Department of Neuroscience, Psychology, Drug Research and Child Health, University of Florence, Florence, Florence Italy; ^3^ Research and Innovation Centre for Dementia‐CRIDEM, AOU Careggi, Florence, Florence Italy; ^4^ Azienda Ospedaliera‐Universitaria Careggi, Florence, Florence Italy; ^5^ IRCCS Fondazione Don Carlo Gnocchi, Florence, Florence Italy

## Abstract

**Background:**

There is an urgent need to move the use of plasma biomarkers from research setting to clinical practice for the early detection of Alzheimer’s disease (AD). The aims of the study were to explore the combined use of plasma p‐tau181 and NfL in Subjective Cognitive Decline (SCD) and Mild Cognitive Impairment (MCI) patients, evaluating diagnostic accuracy and concordance to propose a flow chart for the clinical applicability.

**Method:**

We included 43 SCD, 41 MCI and 21 AD‐dementia (AD‐d) patients, who underwent plasma p‐tau181 and NfL analysis with SiMoA assay. Twenty‐eight SCD, 41 MCI and 21 AD‐d patients underwent CSF biomarkers analysis (Aβ1‐42, Aβ1‐42/1‐40, p‐tau, t‐tau) and were classified as carriers of AD pathology (AP+) it they were A+/T+ (regardless of N), or non‐carriers (AP‐) when they were A‐ (regardless of T and N), A+/T‐/N‐, or A+/T‐/N+ according to the A/T(N) system.

**Result:**

Plasma p‐tau181 and NfL separately showed a good accuracy (AUC=0.88), while the combined model (NfL+p‐tau181) showed an excellent accuracy (AUC=0.092) in discriminating AP+ from AP‐ patients in SCD and MCI. Plasma p‐tau181 and NfL presented only a moderate concordance (Coehn’s k=0.50, p<0.001) in discriminating AP+ from AP‐ patients. Concordant negative cases showed 10.25% of false negativity, concordant positive cases presented 6.66% of false positivity. The only NfL+/p‐tau181‐ patient was AP+. In NfL‐/p‐tau181+, 57.14% of cases were AP‐. A logistic regression analysis showed that both p‐tau181 and NfL significantly influenced the risk of having AD pathology. Using the regression coefficients associated with the two covariates in the logistic model (p‐tau181 and NfL), we estimated the risk of AD pathology: 10.91% (95%C.I. 3.55‐18.27) if both p‐tau181 and NfL were negative; 41.10% (95%C.I. 29.49‐52.71) and 76.49% (95%C.I. 66.48‐86.50) if only one biomarker was positive (respectively p‐tau18 and NfL); 94.88% (95%C.I. 89.69‐100) if both p‐tau181 and NfL were positive.

**Conclusion:**

Considering the moderate concordance and the risk of presenting an underlying AD pathology according to the positivity of plasma p‐tau181 and NfL, the combined use of plasma p‐tau181 and NfL may give added value for the correct interpretation and the detection of AD pathology.